# Associations of Prediabetes, Diabetes and Glucose‐Related Markers With Cognition and Neuroimaging in a 2‐Year Multidomain Lifestyle Randomised Controlled Trial

**DOI:** 10.1002/dmrr.70053

**Published:** 2025-06-06

**Authors:** Thais Lorenzo, Tiia Ngandu, Jenni Lehtisalo, Riitta Antikainen, Juan Domingo Gispert, Nina Kemppainen, Tiina Laatikainen, Jaana Lindström, Juha Rinne, Hilkka Soininen, Timo Strandberg, Rafael de la Torre, Jaakko Tuomilehto, Alina Solomon, Miia Kivipelto

**Affiliations:** ^1^ Neuroimaging Research Group BarcelonaBeta Brain Research Centre Barcelona Spain; ^2^ Division of Clinical Geriatrics Center for Alzheimer Research NVS Karolinska Institutet Stockholm Sweden; ^3^ Neuroimaging of neurodegenerative Diseases and Healthy Aging Neuroscience Research Program Hospital Del Mar Research Institute Barcelona Spain; ^4^ Department of Medicine and Life Sciences Universitat Pompeu Fabra Barcelona Spain; ^5^ Department of Public Health Finnish Institute for Health and Welfare Helsinki Finland; ^6^ Institute of Public Health and Clinical Nutrition University of Eastern Finland Kuopio Finland; ^7^ Center for Life Course Health Research Geriatrics University of Oulu Oulu Finland; ^8^ Medical Research Center Oulu Oulu University Hospital Oulu Finland; ^9^ Centro de Investigación Biomédica en Red Bioingeniería Biomateriales y Nanomedicina (CIBERBBN) Madrid Spain; ^10^ Division of Clinical Neurosciences Turku University Hospital Turku Finland; ^11^ Turku PET Centre University of Turku Turku Finland; ^12^ Wellbeing Services County of North Karelia Joensuu Finland; ^13^ Department of Neurology University of Eastern Finland Kuopio Finland; ^14^ Helsinki University Hospital Helsinki Finland; ^15^ Integrative Pharmacology and Systems Neurosciences Research Group Neurosciences Research Program Hospital Del Mar Research Institute Barcelona Spain; ^16^ CIBER de Fisiopatología de La Obesidad y Nutrición Instituto de Salud Carlos III Madrid Spain; ^17^ South Ostrobothnia Central Hospital Seinäjoki Finland; ^18^ Department of Public Health University of Helsinki Helsinki Finland; ^19^ Saudi Diabetes Research Group King Abdulaziz University Jeddah Saudi Arabia; ^20^ Department of International Health Instituto de Salud Carlos III Madrid Spain; ^21^ Ageing Epidemiology (AGE) Research Unit School of Public Health Imperial College London London UK

**Keywords:** clinical trial, cognition, dementia, dysglycaemia, HbA1c, neuroimaging, oral glucose tolerance test, prediabetes, prevention, risk factors

## Abstract

**Aims:**

Few longitudinal studies have explored Oral Glucose Tolerance Test markers (OGTT) and both cognitive and brain changes. We investigated OGTT and other glycaemia and insulin resistance markers, and cognitive and neuroimaging changes in the Finnish Geriatric Intervention Study to Prevent Cognitive Impairment and Disability (FINGER).

**Materials and Methods:**

At‐risk individuals aged 60–77 years without dementia (*N* = 1259) were randomly enrolled in a 2‐year multidomain lifestyle intervention or regular health advice program. 1025 participants without previously diagnosed diabetes underwent OGTT. Brain MRI scans were available for 132 participants and amyloid (PiB)‐PET and FDG‐PET scans for 47. Cognition was assessed using the modified Neuropsychological Test Battery (mNTB).

**Results:**

Higher baseline dysglycaemia measures, particularly those from the OGTT, were connected to less favourable changes in multiple cognitive measures and hippocampal volume. Higher baseline triglyceride‐glucose (TyG) index was associated with higher amyloid accumulation and decline in brain glucose metabolism. Higher baseline glycated haemoglobin (HbA1c) was related to favourable changes in processing speed and cortical thickness. There were no significant intervention‐control differences in the change in glycaemia markers. Baseline dysglycaemia and glycaemia‐related markers did not modify the previously reported intervention benefits on cognition.

**Conclusions:**

Higher baseline dysglycaemia measures are linked to more deleterious changes in cognition. Specifically, OGTT measures may be the most sensitive for detecting subtle glycaemic abnormalities associated with both unfavourable cognitive and neuroimaging changes. However, HbA1c shows mixed associations with cognition and neuroimaging in people at risk of dementia without previously diagnosed diabetes. This study emphasises the importance of more accurate glucose‐related markers when investigating early stages of glucose metabolism abnormalities and their relationship to subtle cognitive impairment and its structural brain correlates.

**Trial Registration:**

ID NCT01041989 https://clinicaltrials.gov

## 1 Introduction

Cognitive decline and dementia are a major public health concerns leading to late‐life disability and dependence. Previous literature [1, 2] has highlighted dysglycaemia, including diabetes and prediabetes, as risk factors for dementia. Both conditions have been linked to poorer cognitive performance [2, 3], brain atrophy, and cerebrovascular pathology [2, 4]. A key unresolved question concerns which glucose‐related markers are best related to both cognitive decline and structural brain changes, especially in people without diabetes. A substantial between‐study variability in dysglycaemia markers and their definition exists. Among the existing dysglycaemia markers, the oral glucose tolerance test (OGTT) is the most sensitive diagnostic test to detect prediabetes and asymptomatic diabetes. However, the most commonly and consistently used single markers in dementia‐related studies are fasting plasma glucose (FPG) and glycated haemoglobin (HbA1c), with higher glycaemia levels linked to an increased dementia risk, even in individuals without diabetes [5, 6]. However, using only these markers may miss up to 50% of individuals with asymptomatic diabetes and prediabetes [7, 8].

Few studies have reported both cognitive and neuroimaging outcomes in association with glucose‐related measures within the same cohort over time. None of the longitudinal studies have identified significant associations between OGTT‐based glucose measures and changes in both brain function and imaging. There has also been variability in whether comprehensive and sensitive cognitive measures are used, and the choice of imaging methods and measures. Thus, based on earlier findings, it is difficult to systematically assess how the impact of dysglycaemia on cognition is underpinned by an impact on brain structure. Most studies examining the interplay of dysglycaemia with both cognition and neuroimaging have so far been cross‐sectional, with only three longitudinal studies [9, 10, 11]. Results have been inconsistent, depending on several study design characteristics.

Studies that were cross‐sectional or included people with diabetes were more likely to report significant associations with unfavourable cognitive and/or neuroimaging measures, for example grey matter (GM) and white matter (WM) atrophy or reduced cerebral blood perfusion [10, 12, 13, 14, 15]. Dysglycaemia has also been longitudinally associated with GM volume decline [10]. In some cross‐sectional studies excluding diabetes or impaired glucose tolerance, higher levels of dysglycaemia markers (e.g. HbA1c, FPG, one‐hour or two‐hour post‐load glucose (1 h‐PG or 2 h‐PG)) were associated with poorer cognition, particularly memory, lower brain volumes, or WM damage [16, 17].

An interesting observation from previous studies excluding people with diabetes is that associations with cognition and/or brain glucose metabolism as measures by ^18^F‐fluorodeoxyglucose (FDG) ‐ positron emission tomography (PET) scans, or with lower brain microstructure integrity, may be age‐dependent, that is, present among middle‐aged adults but less strong or absent at older ages [9, 18]. In addition, prediabetes, but not diabetes, was cross‐sectionally linked to Alzheimer's disease (AD) PET biomarker (higher brain amyloid accumulation on Pittsburgh Compound B (PiB)‐PET scans) in late middle age, but not at older ages [12]. The impact of glucose metabolism on cognition and the brain has also been suggested to differ between normal ageing and neurodegenerative diseases, although the results are conflicting [11, 19, 20].

The present study investigates associations of dysglycaemia and multiple continuous glycaemia‐related markers with (i) overall cognition, memory, processing speed and executive functioning; and (ii) neuroimaging measures including structural MRI (hippocampal and total GM volumes, cortical thickness, WMH volume), brain amyloid accumulation on PiB‐PET scans, and brain glucose uptake on FDG‐PET, a general marker of brain health highly related with cognition, in the Finnish Geriatric Intervention Study to Prevent Cognitive Impairment and Disability (FINGER) cohort. The FINGER study evaluated the effects of a 2‐year multidomain lifestyle intervention compared with regular health advice on cognition in an older general population at risk of dementia. Findings indicated that the intervention could enhance or preserve cognitive functioning, while also positively impacting health‐related lifestyle factors [21].

We hypothesise that OGTT‐related measures may be more sensitive to deleterious cognitive and brain changes associated with altered glucose metabolism. For this aim, we include the OGTT 2 h‐PG and the PG‐AUC, which has been suggested to be more sensitive by capturing the whole glucose response trajectory, but has been rarely investigated in association with cognition and neuroimaging. For comparison, we also included FPG, HbA1c, insulin, and several indices of insulin resistance: homoeostatic model assessment for insulin resistance (HOMA‐IR), a HOMA2‐IR version accounting for variations in hepatic and peripheral glucose resistance, and the triglyceride glucose (TyG) index as a measure of broader metabolic status beyond glucose metabolism.

## 2 Materials and Methods

### 2.1 Study Design

The FINGER trial is a 2‐year multidomain RCT comprising 1259 participants from the general population from 6 different sites in Finland. The study and main findings have been previously described in detail [21] (Supporting Information S1). The FINGER was approved by the coordinating ethics committee of the Hospital District of Helsinki and Uusimaa.

### 2.2 Participants

The target population was aged 60–77 years and had a Cardiovascular Risk Factors, Ageing and Dementia (CAIDE) risk score 6 points or higher. Participants had cognitive performance scores on the Consortium to Establish a Registry for Alzheimer's Disease (CERAD) neuropsychological battery equal or slightly lower than expected for their age, according to Finnish population norms. Exclusion criteria were dementia or substantial cognitive impairment, and health conditions preventing safe engagement in the intervention. Participants gave written informed consent.

### 2.3 Randomisation and Masking

Participants were randomised in a 1:1 ratio to either a multidomain lifestyle intervention or a control group. Computer‐generated allocation was done in blocks of four (two individuals randomly allocated to each group) at each site after baseline by the study nurse. A total of 631 participants were included in the intervention group, and 628 in the control group. Outcome assessors were blinded to group allocation.

### 2.4 Procedures

The 2‐year multidomain intervention combined nutrition advice, exercise, cognitive training and social activities, and monitoring of vascular/metabolic risk factors as previously described [21]. The control group received regular health advice.

### 2.5 Outcomes

Participants underwent an extensive neuropsychological assessment at baseline, 12 and 24 months. The primary cognitive outcome was a composite Z score based on the modified Neuropsychological Test Battery (mNTB, 14 tests), standardised to the baseline mean and standard deviation (SD). Secondary cognitive outcomes included mNTB domain scores for memory (6 tests), executive functioning (5 tests), and processing speed (3 tests), as previously described [21]. An abbreviated memory score was additionally calculated based on four of the six memory tests with higher complexity.

The FINGER neuroimaging sub‐study was exploratory and included scans conducted at three out of six trial sites. The study design and protocol were previously described in detail (Supporting Information S1). Participants were the most recently recruited individuals at the time when neuroimaging resources became available at each site, and with no contraindications for MRI/PET. Different MR systems were used (1.5 T Avanto Siemens at 2 sites, and 3T Ingenuity Philips at one site). Each site used the same scanner and imaging parameters for both baseline and 2‐year scans. MRI scans were available for 132 participants at baseline, and 112 had repeated 24‐month scans. PiB‐ and FDG‐PET scans were conducted at a single site at baseline (48 participants) and 24 months (39 participants). To measure total GM volume, hippocampal volume, and cortical thickness, Freesurfer (version 5.3: http://surfer.nmr.mgh.harvard.edu/) was used. AD signature regions were selected for computing a cortical thickness measure calculated as the mean of the bilateral entorhinal, inferior temporal, middle temporal, and fusiform areas. WMH volumes were calculated from FLAIR images using a previously described method for WMH segmentation. PiB‐PET and FDG‐PET composite scores were calculated by averaging across bilateral prefrontal, parietal, lateral temporal, anterior cingulate, posterior cingulate and praecuneus areas, as previously described (Supporting Information S1).

Fasting venous blood samples were collected at baseline, 12 and 24‐month visits. Protocols for sample collection, management and glucose‐related measurements were previously described (Supporting Information S1). FPG was measured in all participants, and a 2‐h 75 g‐OGTT with PG measurements at 30 min (30 min‐PG) and 2 h (2 h‐PG) was carried out in participants without diagnosed diabetes. FPG and 2 h‐PG were secondary outcomes as previously published [21]. PG‐AUC was computed using the trapezoidal rule. HbA1c was measured in all participants using immunoturbidimetric method. Serum fasting insulin was measured in a sub‐population of the first 812 participants with fasting blood samples available at both baseline and 24‐month visits, using the multiplex suspension array system Bio‐Plex Luminex 200 instrument (Bio‐Rad Laboratories, Hercules, CA, USA), as part of the Bio‐Plex Pro Human Diabetes 10‐plex panel. HOMA‐IR was calculated from insulin in mU/L and FPG by the previously described formula: [(FPG x insulin)/22.5]. For the calculation of HOMA2‐IR and beta cell function index (HOMA2‐β), we used the HOMA2 Calculator (version 2.2: https://homa‐calculator.informer.com/2.2/). TyG index was computed using the formula [ln (FPG x Triglycerides)/2]. Diabetes at baseline was defined as having a previous diagnosis, glucose‐lowering drug treatment, or FPG ≥ 7.0 mmol/L, or 2 h‐PG in OGTT ≥ 11.1 mmol/L, or HbA1c ≥ 6.5% (cut‐offs according to the Finnish Current Care Guidelines 2020), or HOMA‐IR ≥ 3. Prediabetes was defined as FPG ≥ 6.1–6.9 mmol/L, or 2 h‐PG ≥ 7.8–11 mmol/L, or HbA1c ≥ 6‐6.4% (HbA1c cut‐off according to the International Expert Committee 2009), and no previous diagnosis of diabetes. Dysglycaemia was defined as having either diabetes or prediabetes.

### 2.6 Statistical Analysis

Analyses conducted for this study are post hoc. Intervention effects on changes in PG‐AUC, HbA1c and TyG were investigated in all FINGER participants using mixed effects regression models with maximum likelihood estimation as a function of randomisation group, time, randomisation group x time, age, sex, study site, and diabetes at baseline (yes/no). Intervention effects on changes in insulin and insulin resistance markers (calculated as the difference between baseline and 24 months) were investigated in all participants with available data using linear regression models including randomisation group, age, sex, study site, and diabetes (yes/no).

Zero‐skewness log‐transformation was applied to all skewed continuous glucose‐related, neuroimaging and cognition variables. All continuous glucose‐related, neuroimaging and cognition variables were also *z*‐scored.

Associations of baseline glucose‐associated variables (prediabetes, diabetes, dysglycaemia, PG‐AUC, 2 h‐PG, FPG, HbA1c, insulin, HOMA‐IR, HOMA2‐IR, HOMA2‐β, and TyG) with cognitive scores (mNTB total, memory, abbreviated memory, executive functioning, and processing speed) were analysed using mixed effects regression models with maximum likelihood estimation. Changes in cognitive scores were analysed as a function of individual baseline glucose‐associated variables, glucose‐associated variable x time, randomisation group, time, randomisation group x time, age, sex, education, study site, and diabetes (yes/no). We report the results from these regression models as estimates (with 95% confidence intervals; CI) and *p*‐values for individual glucose‐associated variables (cross‐sectional associations with cognition at baseline) and the glucose‐associated variable × time interactions (longitudinal associations with change in cognition over 2 years). Analyses of prediabetes and diabetes included all FINGER participants. Analyses of individual glucose‐associated variables included FINGER participants who had undergone the OGTT, thus excluding people with previously diagnosed diabetes. Mixed effects regression models with maximum likelihood estimation were used to investigate the potential impact of baseline glucose‐associated variables on the intervention effect on cognition. Changes in cognitive scores were analysed as a function of randomisation group, time, glucose‐associated variable, their 3‐way interaction (group x time x glucose variable), 2‐way interactions (group x time, glucose variable x time, group x glucose variable), age, sex, education, study site, and diabetes (yes/no). We reported results for the 3‐way interaction group x time x glucose variable if estimates (95% CIs) indicated statistically significant effects.

Associations of glucose‐associated variables with neuroimaging measures (hippocampal volume, AD‐signature cortical thickness, total GM volume, WMH volume, PiB‐PET and FDG‐PET composite scores) at baseline were tested using linear regression models with imaging measures as dependent variables, and adjusted for age, sex, diabetes (yes/no), and study site (i.e. MRI scanner type; PET scans were conducted at a single site). Models for volumetric MRI measures were additionally adjusted for estimated total intracranial volume.

Percentage change in imaging measures was calculated as the difference between 2‐year and baseline values, divided by the baseline value and multiplied by 100. Associations of baseline glucose‐associated variables with changes in neuroimaging measures were tested using linear regression models with %‐change in imaging measures as dependent variables, and adjusted for age, sex, randomisation group and diabetes (yes/no). Models for MRI measures were additionally adjusted for the study site and estimated total intracranial volume. Results from all linear regression models were reported as standardised β‐coefficients and *p*‐values.

The level of statistical significance was considered as < 5% in all analyses, which were conducted using Stata version 14 (Stata Statistical Software: Release 14. College Station, TX: StataCorp LP)

## 3 Results

Baseline characteristics of the entire study population (*N* = 1259) as well as the OGTT population (*n* = 1025) are summarised in Table 1. Of those, 47% were women, the mean age was 69 years, a mean of 10 years of education and the mean Mini‐Mental State Examination (MMSE) score of 26.7 points. Supporting Information S1: Table S1 shows baseline characteristics by randomisation group, with no statistically significant differences between the groups. Characteristics of the neuroimaging population with OGTT are shown in Supporting Information S1: Table S2, with no significant differences between intervention and control groups except a somewhat higher baseline FDG‐PET composite score in the control compared with the intervention group. Comparisons between participants with and without available cognitive data at the 2‐year visit, and those with and without neuroimaging data are shown in Supporting Information S1: Tables S3 and S4. Out of the 1025 participants in the OGTT population, mNTB data were available for 950 participants at the 1‐year visit, and 914 at the 2‐year visit.

**TABLE 1:** Baseline population characteristics.

Baseline characteristics	Entire FINGER population (*N* = 1259)		OGTT population (*n* = 1025)	
	*N*	Mean (sd)	*n*	Mean (sd)
Female (%)	587 (47)	—	483 (47)	—
Age	1259	69 (4.7)	1025	69 (4.7)
Education (years)	1258	10 (3.4)	1025	10 (3.4)
MMSE	1255	26.7 (2.1)	1022	26.7 (2.1)
Normal glucose (%)	515 (41)	—	483 (47)	—
Prediabetes (%)	388 (31)	—	358 (35)	—
Diabetes (%)	355 (28)	—	184 (18)	—
FPG (mmol/L)	1256	6.1 (0.9)	1025	5.9 (0.6)
30 min‐PG (mmol/L)	1028	9.1 (1.5)	1025	9.1 (1.5)
2 h‐PG (mmol/L)	1084	7.0 (2.2)	1025	7.1 (2.2)
PG‐AUC	1025	15.9 (2.8)	1025	15.9 (2.8)
HbA1c (%)	1237	5.6 (0.6)	1016	5.5 (0.4)
Insulin (mU/L)	811	6.9 (5.9)	663	6.2 (3.7)
Triglycerides (mmol/L)	1255	1.4 (0.6)	1024	1.3 (0.6)
HOMA‐IR	811	1.9 (2.0)	663	1.7 (1.1)
HOMA2‐IR	811	0.9 (0.8)	663	0.8 (0.5)
HOMA2‐*β*	811	58.2 (30.2)	663	57.5 (23.2)
TyG	1255	8.7 (0.4)	1024	8.7 (0.4)
Cognitive end points (Z scores)				
mNTB total score	1258	−0.006 (0.6)	1025	0.001 (0.6)
Memory	1258	−0.000 (0.7)	1025	0.002 (0.7)
Abbreviated memory	1236	0.002 (0.8)	1006	−0.004 (0.8)
Executive function	1257	−0.016 (0.7)	1024	−0.014 (0.7)
Processing speed	1258	−0.001 (0.8)	1025	−0.022 (0.8)

*Note:* Values are means (SD) unless other specified.

Abbreviations: 30 min‐PG = 30‐min post‐load plasma glucose; 2 h‐PG = 2‐h post‐load plasma glucose; FPG = fasting plasma glucose; HOMA‐IR = homoeostatic model assessment‐insulin resistance index; mNTB = modified Neuropsychological Test Battery; OGTT = oral glucose tolerance test; PG‐AUC = plasma glucose‐ Area under the curve; TyG = triglyceride‐glucose index.

There were no statistically significant associations of baseline prediabetes, diabetes, their combination (dysglycaemia), or any glucose‐ or insulin‐related marker with the intervention effect on cognitive end‐points (randomisation group x time x glucose‐related marker interactions *p* > 0.1, results not shown). No significant differences between the intervention and control groups were found for the change in glucose‐ or insulin‐related markers over 2 years (results not shown).

### 3.1 Associations With Cognitive Performance

Supporting Information S1: Table S5 shows the cross‐sectional and longitudinal associations between prediabetes, diabetes and cognitive scores. At baseline, compared with the normal glucose group, people with prediabetes had lower scores in all cognitive domains (all associations were statistically significant (*p* < 0.05), except for a trend for processing speed (*p* = 0.09)). The diabetes group had significantly lower scores in all cognitive domains, except for non‐significant associations with the two memory scores. Results were similar for dysglycaemia, i.e. inverse associations were observed for all cognitive domains, although this did not reach statistical significance for abbreviated memory (*p* = 0.17), and showed a trend for memory (*p* = 0.08).

In longitudinal analyses, people with prediabetes, diabetes and their combination at baseline were associated with less favourable changes in mNTB total score and memory over 2 years compared with the group without dysglycaemia (Supporting Information S1: Table S5). There was a similar trend for prediabetes and diabetes separately in relation to change in abbreviated memory performance; this was significant when combining the two groups (*p* = 0.02). A trend for less favourable changes in processing speed was also found for the combination of prediabetes and diabetes (*p* = 0.09). No statistically significant longitudinal associations were found with executive functioning.

Table 2 shows cross‐sectional and longitudinal associations between baseline glucose markers and cognitive endpoints. At baseline, higher PG‐AUC, FPG and 2 h‐PG were all associated with a significantly lower mNTB total score. A similar pattern was found for executive functioning, except for FPG showing a trend (*p* = 0.09). Only higher PG‐AUC was significantly related to slower processing speed (*p* = 0.03). An inverse trend was also found for higher PG‐AUC and FPG (but not 2 h‐PG) in relation to lower memory scores. HbA1c was not associated with cognition at baseline.

**TABLE 2:** Associations of baseline glucose markers with cognitive end points (FINGER OGTT population, *N* = 1025).

Cognitive end point	PG‐AUC	FPG	2 h‐PG	HbA1c
	Estimate (95% CI); *p*‐value			
Cross‐sectional at baseline				
mNTB total score	**−0.059 (−0.091 to 0.027);** **<** **0.001**	**−0.034 (−0.067 to −0.002); 0.04**	**−0.047 (−0.078 to −0.015); 0.004**	−0.003 (−0.033 to 0.028); 0.87
Memory	*−0.037 (−0.077 to 0.002); 0.06*	*−0.037 (−0.076 to 0.002); 0.06*	−0.026 (−0.064 to 0.013); 0.20	0.012 (−0.025 to 0.049); 0.51
Abbreviated memory	*−0.038 (−0.081 to 0.005); 0.09*	*−0.041 (−0.084 to 0.001); 0.06*	−0.034 (−0.076 to 0.008); 0.12	0.013 (−0.028 to 0.053); 0.54
Executive functioning	**−0.088 (−0.128 to −0.049);** **<** **0.001**	*−0.034 (−0.074 to 0.005); 0.09*	**−0.075 (−0.114 to −0.036); <** **0.001**	−0.012 (−0.049 to 0.026); 0.53
Processing speed	**−0.053 (−0.100 to −0.005); 0.03**	−0.026 (−0.073 to 0.021); 0.28	−0.039 (−0.086 to 0.007); 0.10	−0.023 (−0.068 to 0.021); 0.30
Longitudinal over 2 years				
mNTB total score	**−0.015 (−0.027 to −0.004); 0.009**	−0.010 (−0.021 to 0.002); 0.10	**−0.021 (−0.033 to −0.010); <** **0.001**	0.000 (−0.011 to 0.012); 0.97
Memory	−**0.027 (−0.045 to −0.008); 0.005**	−0.014 (−0.032 to 0.005); 0.14	**−0.032 (−0.051 to −0.014); 0.001**	−0.005 (−0.024 to 0.013); 0.58
Abbreviated memory	*−0.020 (−0.039* to *−0.000); 0.05*	−0.010 (−0.030 to 0.009); 0.30	**−0.021 (−0.041 to −0.001); 0.04**	0.006 (−0.014 to 0.026); 0.55
Executive functioning	0.000 (−0.014 to 0.015); 0.96	−0.006 (−0.021 to 0.008); 0.39	−0.009 (−0.023 to 0.006); 0.26	−0.000 (−0.015 to 0.014); 0.98
Processing speed	*−0.013 (−0.029 to 0.002); 0.08*	−0.002 (−0.017 to 0.013); 0.84	**−0.020 (−0.035 to −0.005); 0.01**	**0.017 (0.002 to 0.032); 0.03**

*Note:* Bold indicates significant *p*‐value. Values are estimates (95% CIs) and *p*‐values from mixed effects regression models with maximum likelihood estimation.

Abbreviations: 2 h‐PG = 2‐h post‐load plasma glucose; FPG = fasting plasma glucose; HbA1c = glycated haemoglobin; mNTB = modified Neuropsychological Test Battery; OGTT = oral glucose tolerance test; PG‐AUC = plasma glucose‐area under the curve.

**FIGURE 1:**
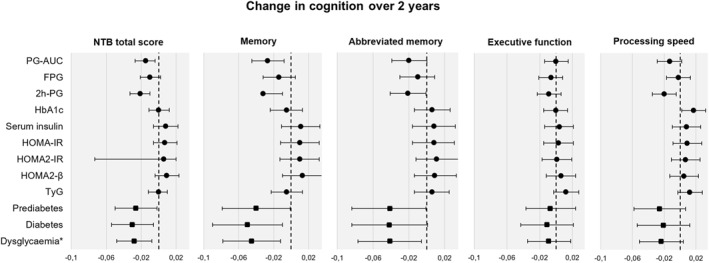
Associations between baseline glucose markers and change in cognition over 2 years. Estimates (95% CIs) are shown from linear mixed models. Negative estimates indicate that higher baseline glucose markers were related to less favourable cognitive changes. Positive estimates indicate that higher baseline glucose markers were related to cognitive improvement. Estimates with 95% CIs that do not cross the dotted reference line are statistically significant. *Dysglycaemia is defined as having either prediabetes or diabetes.

In longitudinal analyses (Figure 1), higher baseline PG‐AUC and 2 h‐PG were associated with less favourable changes in all cognitive domains except executive functioning. Associations for 2 h‐PG were consistently significant across cognitive domains, while PG‐AUC showed a trend for abbreviated memory and processing speed (Table 2). Baseline FPG and HbA1c were not associated with a change in cognition over time, except for processing speed, where a higher performance was associated with higher HbA1c. Further adjustment for plasma creatinine concentration did not modify this result.

Table 3 shows cross‐sectional and longitudinal associations of baseline serum insulin and insulin resistance markers with cognitive endpoints. In cross‐sectional analyses, higher baseline insulin, HOMA‐IR, and HOMA2‐IR were significantly associated with a lower mNTB total score, executive functioning, and processing speed, but not with memory scores. HOMA2‐β had no association with any cognitive score. Higher TyG was associated with lower processing speed score, but not with other cognitive measures at baseline. There were no significant longitudinal associations of baseline serum insulin and insulin resistance markers with changes in cognition over time (Figure 1).

**TABLE 3:** Associations of baseline insulin and insulin resistance markers with cognitive end points (FINGER OGTT population with available data).

Cognitive end point	Serum insulin (*N* = 663)	HOMA‐IR (*N* = 663)	HOMA2‐IR (*N* = 663)	HOMA2‐β (*N* = 663)	TyG (*N* = 1024)
	Estimate (95% CI); *p*‐value				
Cross‐sectional at baseline					
mNTB total score	**−0.045 (−0.085 to −0.006); 0.02**	**−0.048 (−0.088 to −0.008); 0.02**	**−0.044 (−0.084 to −0.005); 0.03**	−0.031 (−0.069 to 0.006); 0.10	−0.010 (−0.040 to 0.021); 0.54
Memory	−0.030 (−0.079 to 0.018); 0.22	−0.031 (−0.080 to 0.018); 0.22	−0.031 (−0.080 to 0.017); 0.21	−0.025 (−0.071 to 0.0203); 0.28	0.026 (−0.011 to 0.062); 0.17
Abbreviated memory	−0.037 (−0.090 to 0.016); 0.17	−0.039 (−0.094 to 0.147); 0.15	−0.032 (−0.081 to 0.017); 0.20	−0.0269 (−0.077 to 0.024); 0.30	0.016 (−0.024 to 0.055); 0.44
Executive functioning	**−0.053 (−0.102 to −0.005); 0.03**	**−0.057 (−0.106 to −0.008); 0.02**	**−0.052 (−0.100 to −0.003); 0.04**	−0.037 (−0.083 to 0.009); 0.12	−0.024 (−0.061 to 0.013); 0.21
Processing speed	**−0.062 (−0.121 to −0.004); 0.04**	**−0.068 (−0.128 to −0.009); 0.03**	**−0.060 (−0.119 to −0.001); 0.05**	−0.038 (−0.093 to 0.018); 0.18	**−0.049 (−0.093 to −0.004); 0.03**
Longitudinal over 2 years					
mNTB total score	0.008 (−0.006 to 0.022); 0.25	0.007 (−0.006 to 0.021); 0.29	0.006 (−0.073 to 0.020); 0.36	0.009 (−0.004 to 0.023); 0.18	−0.000 (−0.012 to 0.010); 0.88
Memory	0.011 (−0.011 to 0.033); 0.34	0.010 (−0.012 to 0.032); 0.38	0.010 (−0.013 to 0.032); 0.40	0.013 (−0.010 to 0.035); 0.27	−0.005 (−0.023 to 0.013); 0.60
Abbreviated memory	0.008 (−0.016 to 0.032); 0.51	0.008 (−0.016 to 0.031); 0.54	0.011 (−0.012 to 0.035); 0.34	0.009 (−0.014 to 0.033); 0.44	0.006 (−0.014 to 0.025); 0.58
Executive functioning	0.004 (−0.014 to 0.021); 0.70	0.003 (−0.015 to 0.021); 0.75	0.001 (−0.017 to 0.019); 0.91	0.006 (−0.012 to 0.024); 0.51	−0.004 (−0.019 to 0.011); 0.59
Processing speed	0.008 (−0.010 to 0.026); 0.38	0.009 (−0.009 to 0.027); 0.34	0.007 (−0.011 to 0.025); 0.44	0.005 (−0.013 to 0.023); 0.56	0.012 (−0.003 to 0.028); 0.11

*Note:* bold indicates significant *p*‐value < 0.05. Values are estimates (95% CIs) and *p*‐values from mixed effects regression models with maximum likelihood estimation.

Abbreviations: HOMA‐IR = homoeostatic model assessment‐insulin resistance index; mNTB = modified Neuropsychological Test Battery; OGTT = oral glucose tolerance test; TyG = triglyceride‐glucose index.

### 3.2 Associations With Neuroimaging Measures

People with dysglycaemia at baseline had a greater decline in hippocampal volume over time compared to the normal glucose group, but no other significant associations with neuroimaging measures were seen cross‐sectionally or longitudinally (Supporting Information S1: Table S6).

Table 4 shows cross‐sectional and longitudinal associations between baseline glucose markers and brain imaging measurements. At baseline, higher PG‐AUC was related to lower cortical thickness, with a trend for a lower total GM volume. Higher FPG was associated with lower total GM and WMH volume, with a trend for lower AD‐signature cortical thickness. 2 h‐PG showed an inverse trend with cortical thickness. HbA1c was inversely associated with the FDG‐PET composite score.

**TABLE 4:** Associations of baseline glucose markers with neuroimaging measures (FINGER neuroimaging population with OGTT, *n* = 115).

Neuroimaging measures	*N*	PG‐AUC	FPG	2 h‐PG	HbA1c	*N*	Serum insulin	HOMA‐IR	HOMA2‐IR	HOMA2‐β	TyG
	Standardised *β* coefficients (*p*‐value)										
Cross‐sectional at baseline											
Hippocampal volume	115	−0.046 (0.63)	−0.092 (0.34)	0.016 (0.86)	0.021 (0.82)	108	**0.204** **(0.03)**	**0.187** **(0.045)**	**0.199** **(0.03)**	**0.235** **(0.007)**	−0.041 (0.64)
Cortical thickness	115	**−0.247** **(0.01)**	*−0.192* *(0.06)*	*−0.191* *(0.06)*	0.094 (0.34)	108	0.034 (0.74)	0.007 (0.94)	0.033 (0.75)	0.108 (0.27)	−0.058 (0.55)
Total GM volume	115	*−0.114* *(0.07)*	**−0.129** **(0.04)**	−0.089 (0.15)	−0.069 (0.26)	108	−0.044 (0.50)	−0.064 (0.33)	−0.042 (0.53)	0.018 (0.78)	**−0.152** **(0.009)**
WMH volume	89	−0.051 (0.64)	**−0.310** **(0.006)**	−0.061 (0.57)	−0.111 (0.30)	83	0.014 (0.90)	−0.026 (0.82)	−0.019 (0.87)	0.124 (0.25)	0.087 (0.41)
PiB‐PET	41	−0.069 (0.69)	0.164 (0.33)	−0.087 (0.61)	−0.099 (0.55)	36	*−0.313* *(0.08)*	−0.295 (0.11)	*−0.332* *(0.06)*	*−0.311* *(0.08)*	−0.029 (0.87)
FDG‐PET	40	−0.204 (0.15)	−0.215 (0.12)	−0.079 (0.59)	**−0.327** **(0.02)**	35	0.204 (0.18)	0.178 (0.24)	0.216 (0.15)	0.238 (0.11)	*−0.258* *(0.07)*
Longitudinal over 2 years											
Hippocampal volume	99	**−0.277** **(0.01)**	**−0.248** **(0.03)**	**−0.269** **(0.01)**	0.070 (0.53)	93	−0.053 (0.64)	−0.082 (0.48)	−0.060 (0.60)	0.036 (0.74)	−0.007 (0.95)
Cortical thickness	99	0.089 (0.42)	−0.105 (0.36)	0.063 (0.57)	**0.216** **(0.048)**	93	−0.016 (0.89)	−0.031 (0.79)	−0.033 (0.78)	0.028 (0.80)	0.161 (0.13)
Total GM volume	99	0.002 (0.99)	−0.157 (0.18)	−0.042 (0.70)	0.138 (0.21)	93	0.038 (0.74)	0.018 (0.88)	0.034 (0.77)	0.093 (0.40)	−0.102 (0.34)
WMH volume	88	0.002 (0.99)	0.023 (0.86)	−0.140 (0.23)	0.179 (0.12)	82	−0.062 (0.60)	−0.055 (0.66)	−0.051 (0.67)	−0.073 (0.52)	0.123 (0.28)
PiB‐PET	33	0.059 (0.74)	0.022 (0.90)	−0.020 (0.91)	−0.126 (0.48)	28	0.063 (0.76)	0.067 (0.75)	0.066 (0.75)	0.052 (0.81)	**0.350** **(0.04)**
FDG‐PET	32	**−0.382** **(0.03)**	**−0.383** **(0.03)**	−0.184 (0.32)	−0.166 (0.36)	27	0.192 (0.32)	0.123 (0.53)	0.161 (0.40)	*0.340* *(0.07)*	**−0.364** **(0.04)**

*Note:* bold indicates significant *p*‐value < 0.05. Values are standardised *β* coefficients (*p* values) from linear regressions.

Abbreviations: 2 h‐PG = 2‐h post‐load plasma glucose; FDG = 18F‐fluorodeoxyglucose; FPG = fasting plasma glucose; GM = grey matter; HbA1c = glycated haemoglobin; HOMA‐IR = homoeostatic model assessment‐insulin resistance index; OGTT = oral glucose tolerance test; PET = positron emission tomography; PG‐AUC = plasma glucose‐area under the curve; PiB = Pittsburgh Compound B; TyG = triglyceride‐glucose index; WMH = white matter hyperintensities.

In longitudinal analyses, higher baseline PG‐AUC, FPG and 2 h‐PG were all associated with reduction of hippocampal volume. Higher PG‐AUC and FPG were also associated with a decline in the FDG‐PET composite score. Baseline HbA1c was inversely related to a lower decline in AD‐signature cortical thickness over time (Table 4).

Associations of serum insulin and insulin resistance markers with neuroimaging measures are also described in Table 4. In cross‐sectional analyses, higher insulin, HOMA‐IR, HOMA2‐IR and HOMA2‐β were related to a higher hippocampal volume at baseline, with insulin, HOMA2‐IR and HOMA2‐β also showing a trend for a lower PiB‐PET composite score. TyG was inversely associated with a baseline total GM volume, with a trend also for the FDG‐PET composite score. In longitudinal analyses, only higher baseline TyG was associated with an increase in PiB‐PET and a decline in FDG‐PET composite scores. Baseline HOMA2‐β tended to be inversely associated with a decrease in the FDG‐PET composite score.

## 4 Discussion

To our knowledge, this is the first comprehensive longitudinal study examining the associations of dysglycaemia and a broad range of glucometabolic markers, including OGTT, with both cognitive function and neuroimaging measures at baseline and longitudinally over 2 years in the same cohort.

Overall, in older individuals at risk for dementia without previously diagnosed diabetes, higher baseline dysglycemia measures were associated with more deleterious changes across multiple cognitive outcomes, with OGTT‐related measures reaching statistical significance. Higher post‐challenge glucose levels in the OGTT and FPG were related to a greater decline in hippocampal volume and brain glucose metabolism. Higher baseline TyG was associated with increased brain amyloid accumulation and greater decline in brain glucose metabolism, which is a novel finding. Interestingly, higher baseline HbA1c was associated with more favourable changes over time in both processing speed and cortical thickness. Consistent with previous reports [21], there were no significant differences between the intervention and control groups regarding changes in glucose‐related markers. Prediabetes, diabetes, and baseline levels of glucose‐related markers did not affect the previously reported intervention benefits on cognition.

Few longitudinal studies have investigated OGTT‐related glucose measures in relation to both cognition and neuroimaging [9, 10, 11], reporting no significant associations. In our study, OGTT‐related measures were consistently associated with changes in both hippocampal volume and overall cognition and memory; processing speed, with 2 h‐PG only. Although FPG and PG‐AUC had similar associations with changes in neuroimaging measures, FPG did not show significant longitudinal associations with cognition. Our findings thus indicate that OGTT‐related markers may be more sensitive than FPG in detecting subtle abnormalities in glucose metabolism associated with unfavourable cognitive changes over time.

Previous literature has reported mixed results regarding FPG and cognition, with cross‐sectional studies showing more likely inverse associations [22], and longitudinal studies more likely to report no associations [2, 23]. Only few longitudinal studies have previously investigated FPG in relation to brain imaging, with results similar to those of this study. Higher FPG was associated with decreasing cortical thickness over 12 years [24], while increasing FPG over 1 year was associated with a decline in hippocampal volume and more brain amyloid accumulation on PET scans [25]. The significance of our cross‐sectional finding of the inverse association between FPG and WMH volume is unclear. Previous results on the association between FPG and WM measures have been mixed, with some studies showing WM microstructural abnormalities in prediabetes [26], while others reported no associations [27]. Since WMH is the hallmark marker of cerebral small vessel disease, its association with glucometabolic markers needs further investigation.

HbA1c is one of the most used single glycaemia parameters in dementia‐related studies, despite having a lower sensitivity for detecting early dysglycaemia compared with the OGTT [7]. In our study, HbA1c showed few and inconsistent associations with cognition and neuroimaging measures in older adults without dementia and previously diagnosed with diabetes. Higher HbA1c was associated with an increase in processing speed and a decrease in cortical thickness longitudinally, but with a lower glucose uptake in the brain on FDG‐PET scans cross‐sectionally. A previous longitudinal study reported an association of higher HbA1c levels with a decrease in GM volume, but not with changes in cognition over time [10]. In two cross‐sectional studies, HbA1c was linked to lower ORs for high amyloid accumulation on PET scans, but not to cognition [12], and to lower total brain volume and lower cognition [28]. These findings were based on cohorts including people with diabetes. In a cross‐sectional study of non‐diabetic older individuals, HbA1c was inversely associated with memory and the volume and microstructure of the hippocampus [16]. Recent reviews [2, 8] have highlighted the heterogeneity of results from studies examining the association between glycaemia parameters with cognition and brain imaging correlates, with many studies reporting no associations, or even positive associations, between FPG or HbA1c and cognitive decline and brain volume.

The variability of results for glucose‐related markers across different studies focussing on cognition and/or neuroimaging may be related to the sensitivity and specificity of the chosen markers. Previous evidence [7] pointed out that HbA1c is a better measurement for monitoring chronic hyperglycaemia in people with diabetes, while OGTT‐related markers are better predictors for insulin resistance, prediabetes, and undiagnosed and incident diabetes. Consequently, it has been suggested that HbA1c and OGTT‐related markers are not interchangeable or fully comparable, and using only HbA1c for diagnosis would miss diabetes and prediabetes cases disproportionately. Instead, the use of OGTT‐related markers for the assessment of glycaemia in non‐diabetic individuals, specifically PG‐AUC, has been suggested as a valuable tool with high significance. It has the potential to enhance the stratification of dysglycaemia risk and may be particularly useful in predicting the impact of dysglycaemia on brain and cognitive function.

Serum insulin and insulin resistance markers were not associated with cognitive decline or change in neuroimaging markers over 2 years in the FINGER study. These markers may thus be less suitable for detecting the impact of subtle abnormalities in glucose metabolism on changes in cognition, especially in people without clinical diabetes. Although several previous studies have reported associations between higher insulin/HOMA indices and cognitive decline, nearly all these studies have also included people with diabetes [8]. Cross‐sectional analyses in our study showed mixed results: associations of higher serum insulin and insulin resistance markers with a lower cognition across several domains, but a higher hippocampal volume. One cross‐sectional study in healthy middle‐aged adults reported that high serum insulin and HOMA‐IR levels were related to poorer cognition and lower total brain (but not hippocampus) volume [28]. The differences in results may be due to the study designs and target populations since the FINGER study included older adults at risk of dementia.

TyG is a marker with potential as an insulin resistance index that is also informative on overall metabolic status and suggested as a better predictor of cardiovascular complications in diabetes than both HOMA‐IR [29] and HbA1c [30]. In our study, TyG was inversely linked with processing speed at baseline but was not longitudinally associated with cognition. However, higher baseline TyG was longitudinally associated with a greater increase in brain amyloid accumulation on PiB‐PET, and a greater decrease in glucose uptake in the brain on FDG‐PET scans, which is a novel finding emphasising the role of metabolic factors in AD‐related brain pathology. Previous studies have suggested that high TyG may be associated with an increased risk of cognitive decline and dementia [31]. Cross‐sectionally, TyG showed an inverse association with GM volume in brain regions involved in AD pathology [32], similar to our cross‐sectional findings.

The main limitations of the study include the use of sub‐populations for serum insulin, insulin resistance markers, and neuroimaging measures. This may have reduced statistical power. Due to these power limitations, results are reported without adjustments for multiple testing and should thus be considered as exploratory. The neuroimaging sub‐study population may not be representative of all FINGER trial participants. MRI scanners differed between study sites; however, this was adjusted for in the analyses. Because the FINGER participants did not have substantial cognitive impairment at baseline, dementia was not a feasible study outcome after 2 years, but an extended follow‐up is ongoing. Although participants are representative of the segment of the Finnish general population at increased risk of dementia, they do not represent the entire risk continuum (from low to high) in the older general population. Since FINGER focused on older adults, this study may not capture some associations previously reported in younger adults.

In conclusion, among older people without diabetes, dysglycaemia markers, particularly those obtained from the OGTT, were associated with changes across multiple cognitive domains and neuroimaging outcomes. The OGTT measures such as 2 h‐PG and PG‐AUC may thus be more sensitive in detecting subtle glucose metabolism abnormalities associated with unfavourable cognitive changes over time. As HbA1c has lower sensitivity for detecting early dysglycaemia compared with OGTT, it may show conflicting associations with cognition and neuroimaging measures in people without dementia and previously diagnosed with diabetes. Our findings emphasise the importance of selecting more accurate glucose‐related markers when investigating the early stages of glucose metabolism abnormalities in relation to subtle cognitive impairment and its structural brain correlates. Accurate glucose‐related markers could also contribute to a more precise selection of individuals at‐risk for dementia who may benefit most from preventive interventions including monitoring and management of dysglycaemia.

## Author Contributions

T.L. (T. Lorenzo), R.A., M.K., T.L. (T. Laatikainen), J.L. (J. Lindström), T.N., J.R., H.S., A.S., T.S., J.T., and RdlT substantially contributed to the conceptualisation of the study. J.L. (J. Lehtisalo) and T.N. contributed to the data curation and directly accessed and verified the data. T.L. (T. Lorenzo) and A.S. directly accessed and verified the data, and executed the formal analysis. T.L. (T. Lorenzo), J.L. (J. Lehtisalo), T.N. and A.S. contributed to the methodology. T.L. (T. Lorenzo), T.N., R.A., T.L. (T. Laatikainen), J.L. (J. Lindström), H.S., T.S., J.T., M.K. and A.S. were involved in funding acquisition. Writing the original draft was in charge of T.L. (T. Lorenzo) and A.S., R.A., J.D.G, N.K., M.K., T.L. (T. Laatikainen), J.L. (J. Lehtisalo), J.L. (J. Lindström), T.N., J.R., H.S., T.S., RdlT and J.T. contributed to the critical review and revision of the manuscript. All authors have read and agreed to the final version of the manuscript for publication.

## Ethics Statement

The FINGER was approved by the coordinating ethics committee of the Hospital District of Helsinki and Uusimaa. Written informed consent was obtained from all participants. This study is registered with ClinicalTrials.gov, number NCT01041989.

## Conflicts of Interest

J.D.G. has received research support from GE HealthCare, Roche Diagnostics and Hoffmann—La Roche, speaker/consulting fees from Biogen, Philips Netherlands, Roche Diagnostics, Esteve and Life‐MI and serves on the Molecular Neuroimaging Advisory Board of Prothena Biosciences; he is the inventor, founder and co‐owner of Betascreen SL. JT owns shares in Orion Pharma, Aktivolabs LTD and Digostics LTD.

## Peer Review

The peer review history for this article is available at https://www.webofscience.com/api/gateway/wos/peer-review/10.1002/dmrr.70053.

## Supporting information

Supporting Information S1

Supporting Information S2

## Data Availability

Public deposition of the deidentified data set is not possible due to legal and ethical reasons, and complete deidentification is not possible as this investigation is part of an ongoing study. The study participants gave informed consent, which includes data use only under confidentiality agreement. Furthermore, the data contain large amounts of sensitive information, and public data deposition may pose privacy concerns. Data dictionary relevant for the present study can be shared upon request by addressing requests to the Finnish Institute for Health and Welfare: kirjaamo@thl.fi. Pseudonymised personal data relevant for the present study can be made available only for those fulfilling the requirements for viewing confidential data as required by the Finnish law and the Finnish Institute for Health and Welfare. Data will be made available only for the purpose of research that is in alignment with informed consent, with investigator support and after approval of a proposal and completion of material transfer agreement.
