# Supplementary material for: Associations of Prediabetes, Diabetes and Glucose‐Related Markers With Cognition and Neuroimaging in a 2‐Year Multidomain Lifestyle Randomised Controlled Trial
Source: Diabetes Metab Res Rev. 2025 Jun 6;41(5):e70053. doi: 10.1002/dmrr.70053 (PMC12143424; doi:10.1002/dmrr.70053)
Supplement: Supplementary file 1 — Supporting Information S1 [file DMRR-41-e70053-s001.docx]

**APPENDIX**

**Table of contents**

**Supplementary text1**

Trial protocol and recruitment process1

Protocol for blood sample collection1

Formula for calculating PG-AUC1

Neuropsychological assessment protocol2

FINGER neuroimaging measures2

FINGER neuroimaging protocol2

MRI assessments3

PET assessments3

**Supplementary references3**

**Supplementary tables5**

**Supplementary text**

**Trial protocol and recruitment process**

The FINGER randomised controlled trial protocol, recruitment process, characteristics of recruited individuals, and comparisons with a relevant reference population population were previosly published.^1,2^ Between Sept 7, 2009, and Nov 24, 2011, 2654 individuals were screened and 1260 were randomly assigned to the multidomain intervention group (n=631) or control group (*n* = 629; 628 after one withdrawal of consent). Overall drop-out rate over two years was 12% (n=153).^2^

Of the 1025 FINGER participants with available OGTT-related data at baseline, 950 underwent the full assessment for the primary cognitive outcome at the 12-month visit, and 914 at the 24-month visit.

**Protocol for blood sample collection**

Protocols for blood sample collection, management and glucose-related measurements were previously described.^2–4^

Fasting blood samples were collected four times during the study at baseline and at six, 12, and 24 months, and laboratory test results were mailed to all participants, together with general written information about the clinical signiﬁcance of measurements, and advice to contact primary health care if needed.^3^ A 2h oral glucose tolerance test with a 75g glucose load was done in the participants without previously diagnosed diabetes. Fluoride citrate tubes were used for glucose samples. The separated serum and plasma samples were frozen immediately and mailed monthly to the laboratory of the National Institute for Health and Welfare, Helsinki, Finland. Plasma glucose concentrations were determined enzymatically using commercial reagents from Abbott Laboratories on a clinical chemistry analyser, Architect c8000 (Abbott Laboratories, Abbott Park, IL, USA)^2^. Insulin was analysed as part of a multimarker set using the multiplex suspension array system Bio-Plex Luminex® 200 instrument, (Bio-Rad Laboratories, Hercules, CA, USA), with the Bio-Plex Pro Human Diabetes 10-plex panel.^4^

***Formula for calculating PG-AUC***^5^

PG-AUC = (FPG + (4 x 30-min glucose) + (3 x 2h-gucose))/4

**Neuropsychological assessment protocol**

The primary outcome of the FINGER trial was the change in overall cognitive performance, evaluated using the total score of an extended version of the Neuropsychological Test Battery (NTB).^6^ The protocol for neuropsychological assessment was previously described.^3,7^

The NTB total score is a composite score derived from 14 cognitive tests, standardised as Z-scores, with higher scores indicating better cognitive performance. Secondary cognitive outcomes focused on domain-speciﬁc NTB z-scores for executive functioning, processing speed, and memory. The executive functioning domain included ﬁve test scores: Category Fluency Test, digit span, Concept Shifting Test (condition C), Trail Making Test (shifting score B – A), and a shortened 40-stimulus version of the original Stroop test (interference score 3 – 2). The processing speed domain included three tests: Letter Digit Substitution Test, Concept Shifting Test (condition A), and Stroop test (condition 2). The memory domain included six test scores: Visual-Paired Associates Test (immediate and delayed recall), Logical Memory Test (immediate and delayed recall), and Word List Memory Test (learning and delayed recall). Additionally, post-hoc analyses were conducted for an abbreviated memory domain that included four of the six tests (two associative memory and two logical memory tests), including extended recall delay (30 min instead of 5 min), requiring more complex processing.

Cognitive assessments were conducted by trained psychologists at baseline, 12 months, and 24 months. Participants who dropped out the study were invited to the ﬁnal assessment at 24 months.

**FINGER neuroimaging measures**

This study focused on six neuroimaging measures with established relevance for Alzheimer’s disease (AD) and dementia:

- Hippocampal volume
- Total grey matter (GM) volume
- Cortical thickness in brain regions typically affected by AD
- White matter hyperintensities (WMH) volume
- Composite measure of brain glucose metabolism on 18F-fluorodeoxyglucose (FDG)- positron emission tomography (PET) scans
- Composite measure of brain amyloid accumulation on Pittsburgh Compound B (PiB)-PET scans

Brain magnetic resonance imaging (MRI) is a critical tool in the diagnostic and prognostic evaluation of dementia-related diseases.^8^ Measures such as hippocampal volume, total grey matter volume, and cortical thickness may be indicative of grey matter atrophy. WMH volume indicates white matter damage and cerebrovascular lesions. FDG-PET measures brain glucose metabolism, providing insight into functional brain activity and its potential decline. PiB-PET detects brain amyloid pathology, a hallmark of AD.^9^ FDG- and PiB-PET are also used in the diagnostic and prognostic evaluation of dementia-related diseases.

**FINGER neuroimaging protocol**^10,11^

The FINGER neuroimaging sub-study was exploratory and included scans conducted at three out of six trial sites.

The study design and protocol were previously described.^12,13^ Participants were the most recently recruited individuals at the time when neuroimaging resources became available at each site, and with no contraindications for MRI/PET. Different MR systems were used, 1.5 T Avanto Siemens (3D-MPRAGE sequence, voxel size 1.2 × 1.2 × 1.2 mm, repetition time (TR) 2400 ms, echo time (TE) 3.5 ms, inversion time (TI) 1000 ms) at the Kuopio and Oulu sites, and 3T Ingenuity Philips (3D turbo field echo sequence [TFE] sequence, voxel size 1.0 × 1.0 × 1.0 mm, TR 8.1 ms, TE 3.7 ms) at the Turku site. Each site used the same scanner and imaging parameters for both baseline and 2-year scans.

***MRI Assessments***

Before quantitative analysis, 3D T1-weighted and fluid-attenuated inversion recovery (FLAIR) images were visually inspected by a neuroradiologist. Participants’ scans were excluded if they had unexpected focal brain lesions and scanning issues potentially impacting volumetry e.g. no full brain coverage, artifacts, intensity inhomogeneity, and inadequate GM/white matter (WM) contrast. At each MRI site, regular phantom scans were performed, and quantitative measures of signal-to-noise ratio, uniformity, and geometric distortion were carried out.

Freesurfer (version 5.3, http://surfer.nmr.mgh.harvard.edu/) was used to measure regional brain volumes and cortical thicknesses. In case of geometric inaccuracy in boundaries between WM, GM, and cerebrospinal fluid (CSF) in the automated WM segmentation, manual editing was conducted. AD signature regions were selected for computing a cortical thickness measure calculated as the mean of the bilateral entorhinal, inferior temporal, middle temporal, and fusiform areas.^14^

WM lesions volume was measured through the segmentation of WMH.^15^ The method is based on the expectation -maximisation algorithm. WML segmentation was done in 3 steps: (i) first, from T1 images, segmentation of WM into 2 classes representing hypointense and normal bright WM regions; (ii) second, using the results of the previous step as initialisation, FLAIR images were segmented into 3 classes: CSF, normal brain tissue, and hyperintense voxels; (iii) third, using the results of the previous initialisation step, WM and subcortical regions were segmented from the FLAIR images in 2 classes. The class with higher intensities was then regarded as the segmentation of WMH.

***PET assessments***

The subsample of FINGER participants from the Turku site underwent ^18^F-FDG dynamic PET scan (GE Advance PET scanner in the 3D scanning mode, General Electric Medical Systems, Milwaukee, WI, USA), and ^11^C-Pittsburgh compound B (PiB)-PET scan (Philips Ingenuity TF PET/MR, Amsterdam, the Netherlands) in addition to structural brain MRI.

For FDG-PET, a dose of ^18^F-FDG 3.7 MBq/kg was injected into an antecubital vein as a bolus with a mean dose of 459 MBq (SD 85 MBq) and flushed with saline. For amyloid PET, on average, 406.3 MBq (SD 107.7 MBq) of ^11^C-PiB-PET was injected intravenously. The scans were quantitatively assessed with the automated region of interest analysis. Composite scores were also calculated: for ^18^F-FDG uptake, the average across the prefrontal, parietal, lateral temporal, precuneus, anterior cingulate, and posterior cingulate cortex; for amyloid deposition, the average across the prefrontal, parietal, lateral temporal, precuneus, anterior cingulate, and posterior cingulate regions of interest.

**Supplementary references**

1. Kivipelto M, Solomon A, Ahtiluoto S, et al. The Finnish Geriatric Intervention Study to Prevent Cognitive Impairment and Disability (FINGER): Study design and progress. *Alzheimer’s and Dementia*. 2013;9(6):657-665. doi:10.1016/j.jalz.2012.09.012

2. Ngandu T, Lehtisalo J, Levälahti E, et al. Recruitment and baseline characteristics of participants in the finnish geriatric intervention study to prevent cognitive impairment and disability (FINGER)—A randomized controlled lifestyle trial. *Int J Environ Res Public Health*. 2014;11(9):9345-9360. doi:10.3390/ijerph110909345

3. Ngandu T, Lehtisalo J, Solomon A, et al. A 2 year multidomain intervention of diet, exercise, cognitive training, and vascular risk monitoring versus control to prevent cognitive decline in at-risk elderly people (FINGER): A randomised controlled trial. *The Lancet*. 2015;385(9984):2255-2263. doi:10.1016/S0140-6736(15)60461-5

4. Pekkala T, Hall A, Mangialasche F, et al. Association of Peripheral Insulin Resistance and Other Markers of Type 2 Diabetes Mellitus with Brain Amyloid Deposition in Healthy Individuals at Risk of Dementia. *Journal of Alzheimer’s Disease*. 2020;76(4):1243-1248. doi:10.3233/JAD-200145

5. JNS Matthews, DG Altman, MJ Campbell, P Royston. Analysis of serial measurements in medical research. *BMJ*. 1990;300:230-235.

6. Harrison J, Minassian SL, Jenkins L, Black RS, Koller M, Grundman M. A Neuropsychological Test Battery for Use in Alzheimer Disease Clinical Trials. *Arch Neurol*. 2007;64(9):1323-1329. doi:10.1001/archneur.64.9.1323

7. Rosenberg A, Ngandu T, Rusanen M, et al. Multidomain lifestyle intervention benefits a large elderly population at risk for cognitive decline and dementia regardless of baseline characteristics: The FINGER trial. *Alzheimer’s and Dementia*. 2018;14(3):263-270. doi:10.1016/j.jalz.2017.09.006

8. Bayram E, Caldwell JZK, Banks SJ. Current understanding of magnetic resonance imaging biomarkers and memory in Alzheimer’s disease. *Alzheimer’s and Dementia: Translational Research and Clinical Interventions*. 2018;4:395-413. doi:10.1016/j.trci.2018.04.007

9. Dubois B, Feldman HH, Jacova C, et al. Research criteria for the diagnosis of Alzheimer’s disease: revising the NINCDS–ADRDA criteria. *Lancet Neurology*. 2007;6(8):734-746. doi:10.1016/S1474

10. Stephen R, Liu Y, Ngandu T, et al. Brain volumes and cortical thickness on MRI in the Finnish Geriatric Intervention Study to Prevent Cognitive Impairment and Disability (FINGER). *Alzheimers Res Ther*. 2019;11(1). doi:10.1186/s13195-019-0506-z

11. Geijselaers SLC, Sep SJS, Stehouwer CDA, Biessels GJ. Glucose regulation, cognition, and brain MRI in type 2 diabetes: A systematic review. *Lancet Diabetes Endocrinol*. 2015;3(1):75-89. doi:10.1016/S2213-8587(14)70148-2

12. Stephen R, Ngandu T, Liu Y, et al. Change in CAIDE Dementia Risk Score and Neuroimaging Biomarkers during a 2-Year Multidomain Lifestyle Randomized Controlled Trial: Results of a Post-Hoc Subgroup Analysis. *Journals of Gerontology - Series A Biological Sciences and Medical Sciences*. 2021;76(8):1407-1414. doi:10.1093/gerona/glab130

13. Sandebring-Matton A, Goikolea J, Björkhem I, et al. 27-Hydroxycholesterol, cognition, and brain imaging markers in the FINGER randomized controlled trial. *Alzheimers Res Ther*. 2021;13(1). doi:10.1186/s13195-021-00790-y

14. Jack CR, Wiste HJ, Weigand SD, et al. Different definitions of neurodegeneration produce similar amyloid/neurodegeneration biomarker group findings. *Brain*. 2015;138(12):3747-3759. doi:10.1093/brain/awv283

15. Wang Y, Catindig JA, Hilal S, et al. Multi-stage segmentation of white matter hyperintensity, cortical and lacunar infarcts. *Neuroimage*. 2012;60(4):2379-2388. doi:10.1016/j.neuroimage.2012.02.034

**Supplementary tables**

**Table S1 |** Baseline population characteristics by randomisation groups

| **Characteristics** | **FINGER population (*N*=1259)** | | | | | | | **FINGER OGTT population (*n*=1025)** | | | | | | |
| --- | --- | --- | --- | --- | --- | --- | --- | --- | --- | --- | --- | --- | --- | --- |
|  | **Intervention (*n*=631)** | | | **Control (*n*=628)** | | | *p* | **Intervention (*n*=513)** | | | **Control (*n*=512)** | | | *p* |
|  | *n* | mean (sd) | | *n* | mean (sd) | |  | *n* | mean (sd) | | *n* | mean (sd) | |  |
| Female, *n* (%) | 286(45) | - |  | 301(48) | - |  | .35 | 231(45) | - |  | 252(49) | - |  | .18 |
| Age | 631 | 69 | (4.6) | 628 | 69 | (4.7) | .28 | 513 | 69 | (4.7) | 512 | 69 | (4.7) | .74 |
| Education (years) | 630 | 10 | (3.5) | 628 | 10 | (3.4) | .92 | 513 | 10 | (3.5) | 512 | 10 | (3.3) | .72 |
| MMSE | 628 | 26.7 | (2.1) | 627 | 26.8 | (2.1) | .64 | 510 | 26.7 | (2.1) | 512 | 26.8 | (2.1) | .30 |
| Normal glucose, *n* (%) | 250(40) | - |  | 265(42) | - |  | .48 | 235(46) | - |  | 248(48) | - |  | .44 |
| Prediabetes, *n* (%) | 204(32) | - |  | 184(29) | - |  |  | 189(37) | - |  | 169(33) | - |  |  |
| Diabetes, *n* (%) | 176(28) | - |  | 179(29) | - |  |  | 89(17) | - |  | 95(19) | - |  |  |
| FPG (mmol/L) | 583 | 6.1 | (0.8) | 600 | 6.2 | (0.9) | .13 | 513 | 5.9 | (0.6) | 512 | 5.9 | (0.6) | .33 |
| 30min-PG (mmol/L) | 514 | 9.1 | (1.5) | 514 | 9.2 | (1.5) | .51 | 513 | 9.1 | (1.5) | 512 | 9.2 | (1.5) | .52 |
| 2h-PG (mmol/L) | 491 | 6.8 | (2.0) | 508 | 7.0 | (2.1) | .08 | 513 | 7.0 | (2.2) | 512 | 7.1 | (2.2) | .94 |
| PG-AUC (mmol/L) | 513 | 15.9 | (2.8) | 512 | 15.9 | (2.7) | .73 | 513 | 15.9 | (2.8) | 512 | 15.9 | (2.7) | .73 |
| HbA1c (%) | 619 | 5.6 | (0.5) | 618 | 5.6 | (0.6) | .73 | 506 | 5.5 | (0.4) | 510 | 5.5 | (0.3) | .75 |
| Insulin (mU/L) | 401 | 7.1 | (6.4) | 410 | 6.7 | (5.4) | .26 | 325 | 6.1 | (3.6) | 338 | 6.2 | (3.8) | .71 |
| Triglycerides (mmol/L) | 630 | 1.4 | (0.6) | 625 | 1.4 | (0.6) | .75 | 513 | 1.3 | (0.6) | 511 | 1.3 | (0.6) | .73 |
| HOMA-IR | 401 | 2.0 | (2.1) | 410 | 1.9 | (2.0) | .33 | 325 | 1.7 | (1.0) | 338 | 1.7 | (1.1) | .97 |
| HOMA2-IR | 401 | 1.0 | (0.8) | 410 | 0.9 | (0.7) | .29 | 325 | 0.8 | (0.5) | 338 | 0.9 | (0.5) | .74 |
| HOMA2-β | 401 | 58.3 | (29.5) | 410 | 58.0 | (30.9) | .88 | 325 | 56.3 | (21.9) | 338 | 58.7 | (24.4) | .20 |
| TyG | 630 | 8.7 | (0.4) | 625 | 8.7 | (0.5) | .62 | 513 | 8.7 | (0.4) | 511 | 8.6 | (0.4) | .52 |
| Cognitive end point (Z scores) | | | | | | | | | | | | | | |
| mNTB total score | 631 | -0.04 | (0.6) | 627 | 0.02 | (0.6) | *.07* | 513 | -0.04 | (0.6) | 512 | 0.04 | (0.6) | **.05** |
| Memory | 631 | -0.03 | (0.7) | 627 | 0.03 | (0.7) | *.08* | 513 | -0.03 | (0.7) | 512 | 0.04 | (0.6) | .11 |
| Abbreviated memory | 622 | -0.03 | (0.8) | 614 | 0.04 | (0.7) | .14 | 506 | -0.03 | (0.8) | 500 | 0.03 | (0.7) | .14 |
| Executive function | 631 | -0.04 | (0.7) | 626 | 0.01 | (0.7) | .27 | 513 | -0.05 | (0.7) | 511 | 0.02 | (0.7) | .15 |
| Processing speed | 631 | -0.04 | (0.8) | 627 | 0.04 | (0.8) | .13 | 513 | -0.03 | (0.8) | 512 | 0.07 | (0.8) | *.05* |

*Notes:* bold indicates significant p-value <0.05; OGTT = oral glucose tolerance test; FPG = fasting plasma glucose; HbA1c = glycated haemoglobin; 30min-PG = 30-minutes post-load plasma glucose; 2h-PG = 2-hour post-load plasma glucose; PG-AUC = plasma glucose- area under the curve; HOMA-IR = homeostatic model assessment- insulin resistance index; TyG = triglyceride-glucose index; mNTB = modified Neuropsychological Test Battery. Values are means (SD) unless otherwise specified.

**Table S2 |** Neuroimaging measures, by randomisation groups

| **Neuroimaging**  **measures** | **FINGER OGTT population (*n*=115)** | | | **Intervention (*n* =59)** | | | **Control (*n* =56)** | | | *p* |
| --- | --- | --- | --- | --- | --- | --- | --- | --- | --- | --- |
|  | *n* | mean (sd) | | *n* | mean (sd) | | *n* | mean (sd) | |  |
| *Baseline* |  |  |  |  |  |  |  |  |  |  |
| ICV (ml) | 115 | 1571.41 | (187.9) | 59 | 1583.83 | (226.14) | 56 | 1558.33 | (270.53) | .47 |
| Hippocampal volume (ml) | 115 | 7.35 | (0.9) | 59 | 7.46 | (0.9) | 56 | 7.23 | (0.9) | .17 |
| Cortical thickness (mm) | 115 | 2.76 | (0.1) | 59 | 2.76 | (0.1) | 56 | 2.77 | (0.1) | .79 |
| Total GM volume (ml) | 115 | 565.04 | (48.0) | 59 | 568.28 | (42.5) | 56 | 561.62 | (53.4) | .46 |
| WMH volume (ml) | 89 | 11.63 | (13.5) | 48 | 11.19 | (11.7) | 41 | 12.18 | (15.5) | .73 |
| PiB-PET composite score | 41 | 1.51 | (0.4) | 21 | 1.48 | (0.4) | 20 | 1.54 | (0.4) | .63 |
| FDG-PET composite score | 40 | 1.22 | (0.1) | 20 | 1.19 | (0.1) | 20 | 1.25 | (0.1) | **.02** |
| *2-year visit* |  |  |  |  |  |  |  |  |  |  |
| ICV (ml) | 99 | 1569.12 | (190.2) | 53 | 1592.19 | (176.0) | 46 | 1542.53 | (204.0) | .20 |
| Hippocampal volume (ml) | 99 | 6.96 | (1.0) | 53 | 7.06 | (1.0) | 46 | 6.85 | (0.9) | .28 |
| Cortical thickness (mm) | 99 | 2.74 | (0.1) | 53 | 2.73 | (0.1) | 46 | 2.75 | (0.2) | .50 |
| Total GM volume (ml) | 99 | 567.54 | (49.1) | 53 | 573.27 | (45.0) | 46 | 560.94 | (53.1) | .22 |
| WMH volume (ml) | 89 | 13.34 | (15.8) | 47 | 13.06 | (14.6) | 42 | 13.66 | (17.2) | .86 |
| PiB-PET composite score | 33 | 1.67 | (0.4) | 16 | 1.66 | (0.5) | 17 | 1.68 | (0.4) | .94 |
| FDG-PET composite score | 33 | 1.20 | (0.1) | 16 | 1.19 | (0.1) | 17 | 1.21 | (0.1) | .46 |

*Notes:* bold indicates significant p-value <0.05; OGTT = oral glucose tolerance test; ICV = estimated total intracranial volume; GM = Grey matter; WMH = White matter hyperintensities; PiB = Pittsburgh Compound B; PET = positron emission tomography; FDG = ^18^F-fluorodeoxyglucose. Values are means (SD).

**Table S3 |** Baseline characteristics of the study population with and without available mNTB data at the 2-year visit

| **Baseline characteristics** | **FINGER OGTT population (*n*=1025)** | | |  | | |  |
| --- | --- | --- | --- | --- | --- | --- | --- |
|  | **With 2-year mNTB (*n*=914)** | | | **Without 2-year mNTB (*n*=111)** | | | *p* |
|  | *N* | mean (sd) | | *n* | mean (sd) | |  |
| Female (%) | 430(47) | - |  | 53(47) | - |  | .89 |
| Age | 914 | 68.7 | (4.7) | 111 | 69.7 | (4.5) | **.02** |
| Education (years) | 914 | 10.0 | (3.4) | 111 | 9.2 | (3.6) | **.02** |
| MMSE | 912 | 26.8 | (2.1) | 110 | 26.4 | (2.2) | *.09* |
| Normal glucose (%) | 433(47) | - |  | 50(45) | - |  |  |
| Prediabetes (%) | 316(35) | - |  | 42(38) | - |  | .79 |
| Diabetes (%) | 165(18) | - |  | 19(17) | - |  |  |
| FPG (mmol/L) | 914 | 5.9 | (0.6) | 111 | 6.0 | (0.6) | **.02** |
| 30min-PG (mmol/L) | 914 | 9.1 | (1.5) | 111 | 9.5 | (1.7) | **.005** |
| 2h-PG (mmol/L) | 914 | 7.0 | (2.2) | 111 | 7.2 | (2.6) | .44 |
| PG-AUC | 914 | 15.8 | (2.7) | 111 | 16.4 | (3.3) | **.03** |
| HbA1c (%) | 905 | 5.4 | (0.4) | 111 | 5.5 | (0.4) | .15 |
| Insulin (mU/L) | 637 | 6.2 | (3.7) | 26 | 5.1 | (2.7) | .12 |
| Triglycerides (mmol/L) | 913 | 1.3 | (0.6) | 111 | 1.3 | (0.7) | .57 |
| HOMA-IR | 637 | 1.7 | (1.1) | 26 | 1.4 | (0.8) | .15 |
| HOMA2-IR | 637 | 0.8 | (0.5) | 26 | 0.7 | (0.4) | .11 |
| HOMA2-β | 637 | 57.8 | (23.4) | 26 | 50.4 | (16.3) | .11 |
| TyG | 913 | 8.7 | (0.4) | 111 | 8.6 | (0.5) | .69 |
| Cognitive end points (Z scores) | 914 |  |  |  |  |  |  |
| mNTB total score | 914 | 0.024 | (0.6) | 111 | -0.194 | (0.6) | **.00** |
| Memory | 897 | 0.022 | (0.7) | 111 | -0.160 | (0.7) | **.01** |
| Abbreviated memory | 913 | 0.018 | (0.7) | 109 | -0.181 | (0.8) | **.01** |
| Executive function | 914 | 0.012 | (0.7) | 111 | -0.228 | (0.7) | **.00** |
| Processing speed | 914 | 0.050 | (0.8) | 111 | -0.205 | (0.8) | **.00** |

*Notes:* bold indicates significant p-value <0.05; OGTT = oral glucose tolerance test; NPS = Neuropsychological scores; FPG = fasting plasma glucose; HbA1c = glycated haemoglobin; 30min-PG = 30-minutes post-load plasma glucose; 2h-PG = 2-hour post-load plasma glucose; PG-AUC = plasma glucose- area under the curve; HOMA-IR = homeostatic model assessment- insulin resistance index; TyG = triglyceride- glucose index. mNTB = modified Neuropsychological Test Battery. Values are means (SD) unless otherwise specified.

**Table S4 |** Baseline characteristics of study population with and without neuroimaging

| **Characteristics** | **FINGER OGTT population (*n*=1025)** | | | | | | | | | | | | | |
| --- | --- | --- | --- | --- | --- | --- | --- | --- | --- | --- | --- | --- | --- | --- |
|  | **Without MRI imaging (*n*=910)** | | | **With MRI imaging (*n*=115)** | | | *p* | **Without PET imaging (*n*=984)** | | | **With PET imaging (*n*=41)** | | | *p* |
|  | *n* | mean (sd) | | *n* | mean (sd) | |  | *n* | mean (sd) | | *n* | mean (sd) | |  |
| Female, *n* (%) | 432(47) | - |  | 51(44) | - |  | .53 | 465(47) | - |  | 18(44) | - |  | .67 |
| Age | 910 | 69 | (4.7) | 115 | 69 | (4.7) | .13 | 984 | 69 | (4.7) | 41 | 70 | (5.1) | .11 |
| Education (y) | 910 | 10 | (3.5) | 115 | 9 | (2.8) | **.03** | 984 | 10 | (3.5) | 41 | 10 | (2.8) | .38 |
| MMSE | 910 | 26.7 | (2.1) | 115 | 27.0 | (1.9) | .16 | 981 | 26.7 | (2.1) | 41 | 27.0 | (1.8) | .42 |
| Normal glucose, *n* (%) | 426(47) | - |  | 57(50) | - |  | .84 | 459(47) | - |  | 24(58) | - |  | .23 |
| Prediabetes, *n* (%) | 319(35) | - |  | 39(33) | - |  |  | 345(35) | - |  | 13(32) | - |  |  |
| Diabetes, *n* (%) | 165(18) | - |  | 19(17) | - |  |  | 180(18) | - |  | 4(10) | - |  |  |
| FPG (mmol/L) | 910 | 5.9 | (0.6) | 115 | 5.9 | (0.7) | .95 | 984 | 5.9 | (0.6) | 41 | 5.8 | (0.7) | .23 |
| 30min-PG (mmol/L) | 910 | 9.1 | (1.5) | 115 | 9.1 | (1.5) | .53 | 984 | 9.1 | (1.5) | 41 | 8.9 | (1.5) | .40 |
| 2h-PG (mmol/L) | 910 | 7.1 | (2.2) | 115 | 6.7 | (1.9) | **.05** | 984 | 7.1 | (2.2) | 41 | 6.5 | (1.5) | .10 |
| PG-AUC (mmol/L) | 910 | 15.9 | (2.8) | 115 | 15.5 | (2.6) | .13 | 984 | 15.9 | (2.8) | 41 | 15.3 | (2.3) | .14 |
| HbA1c (%) | 901 | 5.5 | (0.4) | 115 | 5.5 | (0.3) | .84 | 975 | 5.5 | (0.4) | 41 | 5.4 | (0.2) | .17 |
| Insulin (mU/L) | 555 | 6.3 | (3.6) | 108 | 5.5 | (3.9) | **.02** | 627 | 6.2 | (3.7) | 36 | 5.5 | (3.7) | .27 |
| Triglycerides (mmol/L) | 910 | 1.3 | (0.6) | 115 | 1.3 | (0.6) | .87 | 984 | 1.3 | (0.6) | 40 | 1.3 | (0.7) | .84 |
| HOMA-IR | 555 | 1.7 | (1.1) | 108 | 1.4 | (1.1) | **.03** | 627 | 1.7 | (1.1) | 36 | 1.4 | (0.9) | .18 |
| HOMA2-IR | 555 | 0.9 | (0.5) | 108 | 0.7 | (0.5) | **.02** | 627 | 0.8 | (0.5) | 36 | 0.7 | (0.5) | .24 |
| HOMA2-β | 555 | 58.5 | (22.7) | 108 | 52.4 | (25.3) | **.01** | 627 | 57.6 | (22.9) | 338 | 56.4 | (28.1) | .76 |
| TyG | 910 | 8.7 | (0.4) | 114 | 8.6 | (0.4) | .84 | 984 | 8.7 | (0.4) | 40 | 8.6 | (0.4) | .85 |
| Cognitive end point (Z scores) | | | | | | | | | | | | | | |
| mNTB total score | 910 | 0.00 | (0.6) | 115 | -0.03 | (0.5) | .51 | 984 | 0.00 | (0.6) | 41 | -0.01 | (0.5) | *.09* |
| Memory | 910 | 0.01 | (0.7) | 115 | -0.08 | (0.6) | .15 | 984 | 0.00 | (0.7) | 41 | -0.06 | (0.6) | .53 |
| Abbreviated memory | 891 | 0.01 | (0.8) | 115 | -0.13 | (0.7) | *.06* | 965 | 0.00 | (0.8) | 41 | -0.15 | (0.7) | .21 |
| Executive function | 909 | -0.02 | (0.7) | 115 | -0.00 | (0.6) | .82 | 983 | -0.02 | (0.7) | 41 | 0.02 | (0.6) | .78 |
| Processing speed | 910 | 0.02 | (0.8) | 115 | 0.02 | (0.8) | .93 | 984 | 0.02 | (0.8) | 41 | 0.06 | (0.8) | .75 |

*Notes:* bold indicates significant p-value <0.05; OGTT = oral glucose tolerance test; FPG = fasting plasma glucose; HbA1c = glycated haemoglobin; 30min-PG = 30-minutes post-load plasma glucose; 2h-PG = 2-hour post-load plasma glucose; PG-AUC = plasma glucose- area under the curve; HOMA-IR = homeostatic model assessment- insulin resistance index; mNTB = modified Neuropsychological Test Battery; TyG = triglyceride- glucose index. Values are means (SD) unless otherwise specified.

**Table S5 |** Associations of baseline prediabetes and diabetes with cognitive end points (entire FINGER population, N=1256)

| **Cognitive end point** | **Prediabetes** | | **Diabetes** | | **Dysglycaemia**  **(prediabetes or diabetes)** | |
| --- | --- | --- | --- | --- | --- | --- |
|  | **Estimate (95% CI), p-value** | | | | | |
| *Cross-sectional at baseline* |  |  |  |  |  |  |
| mNTB total score | **-0.101 (-0.165 - 0.037);** | **0.002** | **-0.071 (-0.136 - -0.006);** | **0.03** | **-0.087 (-0.141 - -0.032);** | **0.002** |
| Memory | **-0.089 (-0.167 - -0.010);** | **0.03** | -0.029 (-0.110 **-** 0.052); | 0.49 | *-0.060 (-0.127 - 0.007);* | *0.08* |
| Abbreviated Memory | **-0.094 (-0.180 - -0.007);** | **0.04** | -0.007 (-0.097 - 0.082); | 0.87 | -0.052 (-0.167 - 0.022); | 0.17 |
| Executive functioning | **-0.129 (-0.206 - -0.051);** | **<0.001** | **-0.083 (-0.162 - -0.003);** | **0.04** | **-0.107 (-0.173 - -0.041);** | **0.002** |
| Processing speed | *-0.082 (-0.175 - 0.122);* | *0.09* | **-0.146 (-0.243 - -0.050);** | **0.003** | **-0.112 (-0.193 - -0.032);** | **0.006** |
| *Longitudinal over 2 years* |  |  |  |  |  |  |
| mNTB total score | **-0.026 (-0.050 - -0.002);** | **0.03** | **-0.030 (-0.054 - -0.006);** | **0.02** | **-0.028 (-0.048 - -0.008);** | **0.007** |
| Memory | **-0.040 (-0.079 - -0.001);** | **0.04** | **-0.050 (-0.090 - -0.010);** | **0.01** | **-0.045 (-0.078 - -0.012);** | **0.008** |
| Abbreviated Memory | *-0.041 (-0.084* **-** *-0.001);* | *0.05* | *-0.042 (-0.084 - 0.001);* | *0.06* | **-0.041 (-0.077 - -0.006);** | **0.02** |
| Executive functioning | -0.007 (-0.037 **-** 0.024); | 0.68 | -0.011 (-0.043 **-** 0.021); | 0.49 | -0.009 (-0.035 **-** 0.018); | 0.52 |
| Processing speed | -0.026 (-0.058 **-** 0.007); | 0.12 | -0.021 (-0.054 **-** 0.012); | 0.21 | *-0.024 (-0.051* **-** *0.004);* | *0.09* |

*Notes:* bold indicates significant p-value <0.05; mNTB = modified Neuropsychological Test Battery. Values are estimates (95% CIs) and p values from mixed effects regression models with maximum likelihood estimation.

**Table S6 |** Associations of baseline prediabetes and diabetes with neuroimaging markers (FINGER neuroimaging population, N=132)

| **Neuroimaging**  **measures** | **N** | **Dysglycaemia (prediabetes or diabetes)** | |
| --- | --- | --- | --- |
|  |  | **Standardised β coefficients**  **(*p*-value)** | |
| *Cross-sectional at baseline* |  |  |  |
| Hippocampal volume | 132 | 0.041 | (0.61) |
| Cortical thickness | 132 | -0.086 | (0.32) |
| Total GM volume | 132 | -0.082 | (0.12) |
| WMH volume | 100 | -0.076 | (0.43) |
| PiB-PET | 48 | -0.057 | (0.71) |
| FDG-PET | 46 | -0.114 | (0.39) |
| *Longitudinal over 2 years* |  |  |  |
| Hippocampal volume | 111 | **-0.238** | **(0.015)** |
| Cortical thickness | 111 | -0.056 | (0.56) |
| Total GM volume | 111 | -0.105 | (0.30) |
| WMH volume | 99 | -0.163 | (0.11) |
| PiB-PET | 39 | 0.124 | (0.46) |
| FDG-PET | 37 | -0.204 | (0.23) |

*Notes:* bold indicates significant p-value <0.05; GM = grey matter; WMH = white matter hyperintensities; PiB = Pittsburgh Compound B; PET = positron emission tomography; FDG = ^18^F-fluorodeoxyglucose. Values are standardised β coefficients (p values) from linear regressions.
