# Supplementary material for: Associations of Prediabetes, Diabetes and Glucose‐Related Markers With Cognition and Neuroimaging in a 2‐Year Multidomain Lifestyle Randomised Controlled Trial
Source: Diabetes Metab Res Rev. 2025 Jun 6;41(5):e70053. doi: 10.1002/dmrr.70053 (PMC12143424; doi:10.1002/dmrr.70053)
Supplement: Supplementary file 2 — Supporting Information S2 [file DMRR-41-e70053-s002.docx]

**Short summary**

Among older people without diabetes, dysglycaemia markers, particularly OGTT-related, were associated with changes across multiple cognitive domains and neuroimaging outcomes. Measures such as 2h-PG and PG-AUC may be more sensitive in detecting subtle glucose metabolism abnormalities associated with unfavourable cognitive changes. As HbA1c has lower sensitivity for detecting early dysglycaemia compared with OGTT, it may show conflicting associations with cognition and neuroimaging measures in people without dementia and previously diagnosed diabetes. Our findings emphasise the importance of selecting accurate glucose-related markers when investigating early stages of glucose metabolism abnormalities in relation to subtle cognitive impairment and its structural brain correlates.
